# Characterization of immune infiltrates, molecular subtypes and signature genes in patients with allergic rhinitis

**DOI:** 10.1515/med-2026-1441

**Published:** 2026-06-01

**Authors:** Wenfei Yang, Ruiqing Wang, Shaoping Peng, Xiao Liu

**Affiliations:** Department of Otorhinolaryngology Head and Neck Surgery, The First Affiliated Hospital of Gannan Medical University, Ganzhou, China

**Keywords:** allergic rhinitis, immune infiltration, diagnostic model, feature genes

## Abstract

**Objectives:**

Allergic rhinitis is a disorder of immune response disease, but to date there still gaps in understanding the molecular characters of allergic rhinitis. This study integrates deconvolution algorithm and machine-learning approach to characterize allergic rhinitis molecular features.

**Methods:**

Functional enrichment showed that negative regulation of phosphate metabolic process and Glycolysis/Gluconegenesis pathway were significantly altered after allergic rhinitis onset. The infiltration levels of T cells memory resting, Monocytes and Macrophage M1 wrere significantly changed between allergic rhinitis and control groups.

**Results:**

Four allergic rhinitis feature genes were screened via four machine-learning approaches and a diagnostic model was constructed based on the feature genes. The differential expression levels between AR and the control groups were validated using an OVA-induced AR mouse model. *In vivo* knockdown of ALDH3B2 significantly alleviated AR symptoms.

**Conclusions:**

Five AR feature genes were obtained with machine-learning algorithms. The AR feature genes were used to construct a diagnostic model. The function of those genes in AR progression was validated *in vivo* using an OVA-induced AR mouse model.

## Introduction

Allergic rhinitis (AR) is a prevalent inflammatory condition affecting the nasal mucosa of people of all ages, but it is most common among teenage [1]. The 12-month prevalence of AR increased fourfold from 6 % (at age 3) to 24 % (at age 13) in children whose parents had no allergies. It also increased threefold from 13 % (at age 3) to 44 % (at age 13) in children with at least one allergic parent. At least 50 % of the children experienced severe AR [1], persistent symptoms. Although boys are more likely than girls to have AR, this tendency reverses during puberty, resulting in equal prevalence among men and women by adulthood. Although AR is not a serious illness it is clinically relevant because it causes many complications and negatively impacts quanlity of life, as well as productivity at work and school. It is estimated that AR affects around 400 million people worldwide, with higher prevalence in industrialized nations [2], [3], [4]. Therefore, there is a pressing need for comprehensive research on allergic rhinitis to identify novel therapeutic targets and improve treatment outcomes.

AR occurs when the epithelial barrier is disrupted, allowing allergens to penetrate the nasal passageway mucosal epithelial. The allergens are then processed by antigen-presenting cells, which cause the differentiation of naive T cells into T-helper type 2 cells. These cells are capable of releasing cytokines [5], 6]. This process promotes B-cell production of allergen specific IgE antibodies, which binds to high-affinity IgE receptors on mast cells, Langerhans cells, monocytes, and basophils [7]. Upon reexposure to the same allergen, the relevant anti-genic determinant is recognized by the IgE antigen-binding site of the IgE molecule, which activate mast cell and cause it to release bioactive mediators such as histamine, leukotriens, and platelet-activating factor. These mediators bind to receptors on blood vessels, mucous-secreting glands, and sensory nerves, that are associated with symptoms of AR [8]. Epithelial cell disruption also releases alarmin cytokines, which further promoting the inflammatory response. Eosinophils are type 2 inflammatory cells that enter the nasal mucosa in response to IL-4 and IL-5 and release cytokines, chemokines, leukotrinens, prostaglandins, and toxic proteins or enzymes perpetuating inflammation. In addition, trigeminal neurons express neuropeptides, cholinergic sensory receptors, and transient response potential receptors that can be activated by mechanical, osmotic, thermal, and chemical stimuli. These neurogenic pathways play an important pathogenic role in mixed and nonallergic rhinitis [8], 9]. Currently, the primary pharmacologic therapies for AR in clinical practice include antihistamines, glucocorticoids, anti-leukotrienes, and mast cell stabilizers [10]. These agents alleviate AR symptoms through distinct mechanisms of action; however, their use may associate with adverse effects in some patients. Those therapeutic strategies for AR primarily focus on alleviating immune-related responses; however, effective prevention or elimination of AR will require a deeper understanding of the underlying mechanisms driving it onset.

Up to half of patients suffering from chronic rhinitis may also experience a condition known as mixed rhinitis. This means their symptoms are triggered by both allergic and nonallergic factors [11]. Certain clinical features help differentiate AR from mixed rhinitis, which is characterized by features of both AR and nonallergic rhinitis (NAR). For example, a questionnaire was distributed to 100 randomly selected patients with chronic rhinitis, multivariable logistic regression analysis and maximum likelihood estimates estimate shows that absence of seasonal outdoor symptoms, absence of a parental history of allergy, absence of symptoms in the presence of cats, presence of symptoms around perfumes and fragmented, and being older than 35 years of age at symptom onset were most associated with NAR [12]. A meta-analysis with 430 patients from Health Canada reported that the skin prick tests had an 85 % sensitivity and 77 % specificity for confirming AR [13]. Despite the high prevalence of AR, current therapies remain symptomatic with limited efficacy in some patients. A systematic characterization of AR’s molecular landscape – integrating immune infiltration patterns, molecular subtypes, and diagnostic biomarkers – is urgently needed to develop targeted therapies and improve diagnostic precision.

We hypothesized that AR exhibits distinct immune infiltration patterns and molecular subtypes, and that machine learning can identify robust feature genes for diagnostic modeling. However, a comprehensive analysis integrating these aspects – immune microenvironment, molecular classification, and biomarker discovery via machine learning – is still lacking. Therefore, the primary rationale of this study was to systemically characterize the immune infiltrates and molecular landscape of AR and to identify reliable diagnostic biomarkers using integrative bioinformatics and machine-learning approaches. Our central research question was: Can we identify distinct molecular subtypes and a set of robust feature genes in AR patients that are associated with specific immune cell infiltration patterns and have diagnostic utility? Through integrative machine learning analysis, five candidate biomarkers were identified for AR diagnosis by this study. Among them, ALDH3B2 knockdown *in vivo* markedly mitigated AR-related symptoms, indicating its promise as a novel therapeutic target.

## Materials and methods

### Data retrieving

The GSE51392 and GSE44037 dataset were downloaed from the Gene Expression Omibus (GEO) database (http://www.ncbi.nlm.nih.gov/geo), then merged and batch effects was removed using the R package sva (v3.54.0) [14]. In addition, principal component analysis (PCA) was employed to evaluate the effect of batch correction. Poly (I:C)-treated samples were included in the analysis to capture broader molecular signatures of AR under heterogeneous environmental stimuli.

### Differential expression analysis

After batch effect correction and normalization with rma algorithm [15], limma (v3.62.2) [16] R package was applied for between group difference analysis, p<0.05 and |log2 Fold Change|>0.585 threshold was used to obtain differential expressed genes (DEGs) between AR and the control groups. Subsequently, volcano plot was used to visualize the differential expression result. Furthermore, we used RCircos (v1.2.2) [17] package to visualize the position of DEGs on chromosomes.

### Correlation analysis of DEGs

To investigate the correlation of expression between DEGs, spearman correlation coefficients was obtained between genes and then the results were visualized with corrplot (v0.95) [18] and circlize (v0.4.16) [19] R packages.

### GO and KEGG enrichment analysis

To gain inside the function of the DEGs, gene ontology (GO) and Kyoto Encyclopedia of Genes and Genomes (KEGG) enrichment analysis using clusterProfiler (v4.14.6) [20] R package was performed, and the results were visualized with ggplot2 (v3.5.1) [21] and circlize (v0.4.16) [19] R package.

### Analysis of immune cell infiltration

The GSE51392 and GSE44037 datasets were analyzed using the CIBERSORT algorithm (http://cibersort.stanford.edu/) to determine the abundance of 22 immune cell subtypes. These subtypes exemplify the cellular composition of the immune microenvironment in AR, and Spearman correlation analysis was conducted on gene expression and immune cell infiltration abundance. And the results were visualized with ggplot2 (v3.5.1) [21] and linkET R packages.

### Consensus clustering analysis

Consistency clustering is a resampling-based approach. It identifies each member and their subgroup number. It also validates the cluster. To discover various subtypes of AR patterns based on the DEGs, the ConsensusClusterPlus (v1.70.0) [22] package in R was employed. The optimal number of clusters k is determined based on the cumulative distribution function (CDF) curve, clustering consensus score and consensus matrix. And linear dimensional reduction was employed to validate the identified subgroups of AR.

### Screening AR feature genes

Our study employed four machine learning methods to select AR feature genes [1]: LASSO regression (glmnet) selected genes with non-zero coefficients at optimal λ (10-fold cross-validation) [2]; SVM-RFE recursively eliminated features with minimal impact on model accuracy [3]; Random Forest (RF) ranked genes by mean Gini impurity reduction [4]; XGBoost (XGB) was optimized via grid search. Final features were genes identified by ≥3 algorithms. The R package glmnet (v4.1.8) [23] was used to determine the optimal penalty value with the least binomial deviation. The R packages e1071 (v1.7.16) [24], kernlab (v0.9.33) [25], and caret (v7.0.1) [26] were used to perform the support vector machine recursive feature elimination (SVM-RFE) method. The result with the minimum cross-validation error was then examined. The randomForest (v4.7.1.2) [27] R package was used to develop the RamdomForest method for determing the level with the least amount of error. Based on Receiver operating characteristic (ROC) and model residuals, the XGB model was choosed and the top five importance genes of this model were selected as AR features genes.

### Animal model and grouping

Six-to eight-week-old male BALB/c mice weighing 18–20 g were obtained from the Shanghai Model Organisms Center, Inc. (Male mice were selected to minimize hormonal variability in immune responses, as estrogen is known to modulate Th2 inflammation) and maintained under specific pathogen-free (SPF) conditions. All the mice were cared for in a quarantine room for one week before the experiment. They were given water and pellet food *ad libitum*. The mice were randomly assigned into four groups: control, ovalbumin (OVA), OVA+LV-shNC, OVA+LV-shALDH3B2. OVA was obtained from Shanhai Shenggong Biotechnology (T510211). The sample size was determined based on preliminary power analysis (α=0.05, power=0.8) using effect sizes from prior AR studies (GSE51392 effect size=1.2), yielding a minimum requirement of 6 mice per group. To account for potential attrition, we included 8 mice per group. On days 1, 7, and 14, the mice in the OVA, OVA+LV-shNC, and OVA+LV-shALDH3B2 groups were injected intraperitoneally with 200 μL of PBS containing 25 μg of OVA and 2 mg of aluminum hydroxide adjuvant, while the control group was injected with only PBS. From days 21 to 25, the mice were given a nasal challenge consisting of 20 ul of OVA solution containing 400 ug of OVA in each nasal cavity once a day. On day 25–27, the mice in the OVA-LV-shNC and OVA-LV-shALDH3B2 were intranasally injected about 1e8 TU shNC and shALDH3B2 expression lentiviral. Three days after the final lentiviral injection, the mice were sacrificed by cervical dislocation after being anesthetized with acepromazine (0.75 mg/kg; A7111, Sigma-Aldrich). After treatment, nasal tissue, blood, and nasal lavage fluid (NAFL) were collected.

### Nasal symptoms

Nasal symptoms were assessed by recording the frequencies of nasal rubbing and sneezing for 30 min following the final intranasal OVA challenge. Observations were performed by blinded investigators.

### RNA extraction and reverse transcription-quantitative PCR (RT-qPCR)

Total RNA was extracted from nasal mucosal tissues of each group of mice using TRIzol reagent (Shenggong Biotech, China), and cDNA was synthesized from the RNA using a PrimeScript RT kit (Takara). The cDNA was then amplified and quantified using a SYBR Green mix (Shengong Biotech) on an Applied Biosystems 7,500 instrument. The cDNA was amplified with the following program: 1. pre-denature at 95 °C for 5 min; 2. denature at 95 °C for 30 s; 3 annealing at 60 °C for 15 s; 4 amplification at 72 °C for 15 s; and repeats the step 2 to step 4 for 39 cycles. The results were calculated with the 2^−ΔΔCT^ method. The primer sequences used as Table 1.

**Table 1:** RT-qPCR primer sequences.

Gene	Forward (5′ to 3′)	Reverse (5′ to 3′)
CRYZ	ACT​CCG​GGT​TCA​CAA​CTT​CC	AGC​CCT​CAT​CAA​CTT​CTG​CC
IGFBP3	CAC​TGC​CCT​CAC​TCT​GCT​C	GCGCGCACTGGGACA
ALDH3B2	CCA​TTC​CGG​TTC​CCT​AGA​GC	GTC​ACA​CTG​CCT​TGC​CCT​AT
LOC645166	ACC​CAT​TCT​GGA​AGT​GTC​TGC	TAT​ACC​TCA​CCC​GTC​TCC​CA
GTF2H2	GGA​GAA​GAA​GCC​TGG​AGC​TG	CCG​CTT​GGT​TCT​CTC​AGG​TT
GAPDH	GAG​CCT​CCT​CCA​ATT​CAA​CCC	GGG​ACG​AGG​AAA​CAC​TCT​CC

### Western blot

After built the AR mouse model, the nasal mucosal tissues were collected. The tissue was lysed and protein quantification was determined by BCA method (Shenggong Biotech). A protein sample of 10–20 µg was loaded onto an 8–30 % acrylamide-bis gel, transferred through a 0.22-µm pore polyvinylidene fluoride (PVDF) membrane, and blocked with 5 % nonfat milk for 1 h at room temperature. Then primary antibody (CRYZ, GTX118270, 1:1000, GeneTex; IGFBP3, 10189-2-AP, 1:4000, proteintech; ALDH3B2, 15746-1-AP, 1:500, proteintech; GTF2H2, 16005-1-AP, 1:500, proteintech; β-actin, NB600-501, 1:5000, Novus Biologicals; GAPDH, 10494-1-AP, 1:5000, proteintech) was added, and incubated at 4 °C overnight. At day 2, the secondary antibodies (554021 BD Pharmingen, 1:1000; 550826, BD Pharmingen, 1:1000) were introduced and the sample was incubated at room temperature for 1 h. Then, the luminescent solution was added for exposure and color development. ImageJ software was used for band intensity analysis, the abundance of proteins was calculated relative to the β-actin and GAPDH.

### Haematoxylin and eosin (H&E) staining

The nasal mucosa tissues were fixed in 10 % neutral formalin (E672001, Shenggong Biotech, China) for at least 24 h, then embedded in paraffin. Then, the tissues were sectioned into slices. After that, the sections were embeded in xylenes for deparaffinization. Tissue samples were rehydrated through a graded ethanol series prepared with 100 % ethanol diluted in double-distilled water. Sections were subsequently rinsed two times with PBS for 3 min each. For histological staining, sections were immersed in haematoxylin solution (C0107, Beyotime, China) for 10 min to visualize nuclei, followed by eosin solution (C0109, Beyotime, China) for 3 min to stain the cytoplasm.

### ELISA assay

The serums of each mice were collected, and OVA-specific IgE concentrations were determined using a mouse OVA-IgE ELISA kit (F10731, Westang, Shanghai, China) following the manufacturer’s instructions. Optical density (OD) was measured at 450 nm using a microplate reader (SpectraMax iD3, Silicon Valley, CA).

Nasal lavage fluid (NALF) samples were removed from storage at −70 °C and thawed at room temperature. The concentrations of IL-4, IL-5, and IL-13 were quantified using commercial ELISA kits (E-EL-H0101, PI625, E-EL-M0727) following the manufacturer’s protocols. Absorbance was measured at 450 nm using a microplate reader.

### Measurement of histamine

The histamine levels present in the serum of mice were ascertained through the implementation of ophthalaldehyde spectrofluorometric analysis. Fluorescent intensity was measured at a wavelength of 44 nm (with an excitation wavelength of nm) using a spectrofluorometer (Agilent Cary Eclipse).

### Construct and validate nomogram

Based on the five AR feature genes, we constructed a diagnosis nomogram for AR with rms (v8.0.0) [28] R package. The calibration curve showed minimal difference between the actual and predicted AR risks. This suggests that the nomogram model for AR is precise. Since the decision curve analysis (DCA) “Model” curve stays consistently higher than both “Treat All” and “Treat None,” this suggests that the predictive model is consistently beneficial, and provides a reliable advantage across all threshold probabilities.

### Statistical analysis

Statistical analyses were performed using SPSS version 23.0 (IBM Corp., Armonk, NY) and R software (v4.4.3). Data were tested for normality and homogeneity of variance, and presented as mean ± SD. Group comparisons were performed using nonparametric t-tests, with p<0.05 considered statistically significant. When the data was found to be non-normal, the Kruskal-Wallis test was used. And spearman’s rank test was used to assess the strength of correlations.

#### Ethics approval and consent to participate

All experiments were approved by the animal ethics committee of the First Affiliated Hospital of Gannan Medical University.

## Results

### Integrate two AR datasets

**Figure 1:**
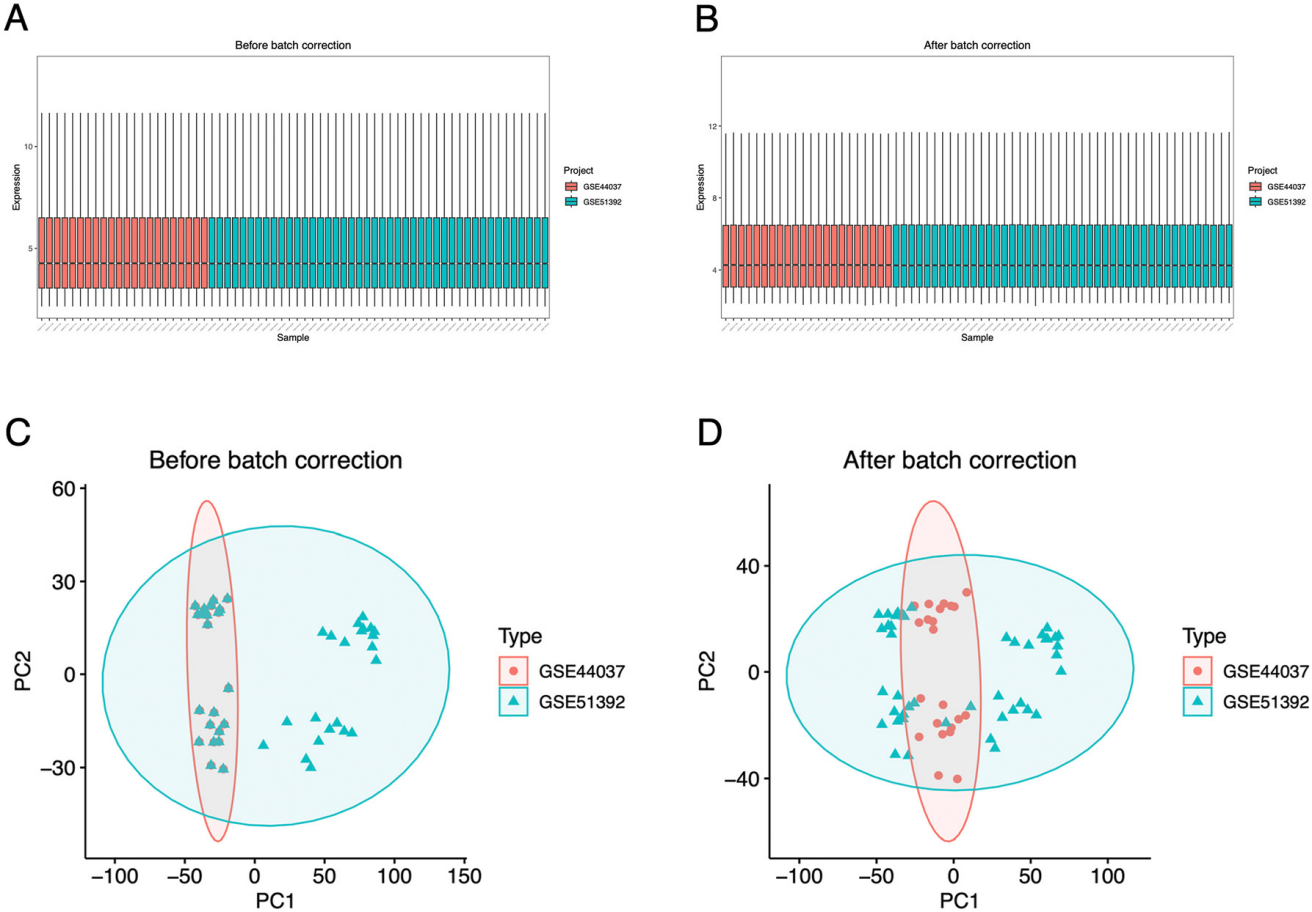
Merge two allergic rhinitis (AR) datasets. (A, B) The expression of all genes in each sample before and after batch correction. (C, D) Principal component analysis (PCA) of all the samples before and after batch correction.

We merged the two datasets GSE51392 and GSE44037 into one cohort (Figure 1A and B), and the merger yielded a total of 30 AR samples, 36 HC samples and 19,919 expressed genes (Table S1). By batch correction and PCA analysis, we found no significant batch effect between the two datasets (Figure 1C and D).

### Differential expression analysis

**Figure 2:**
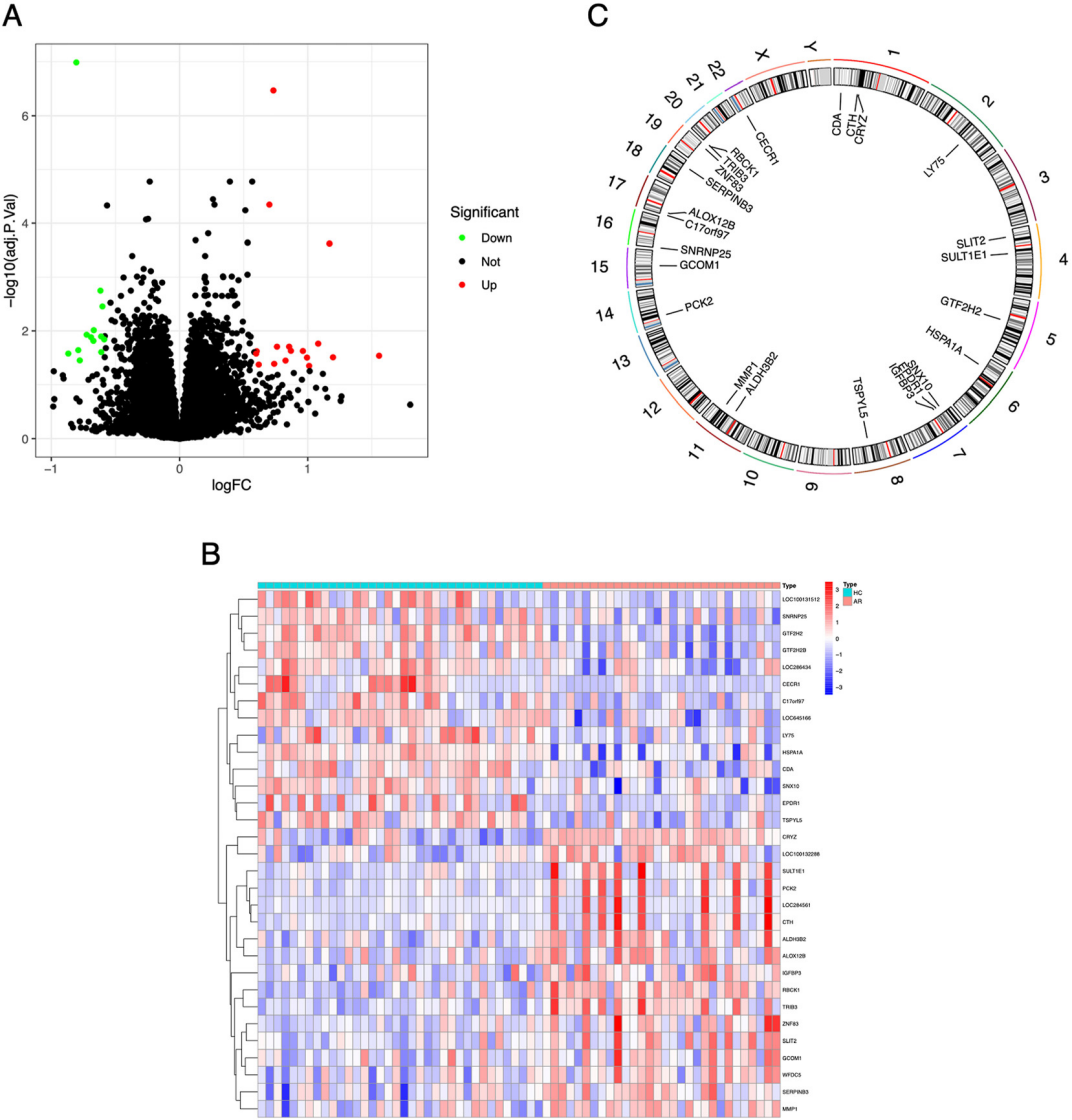
Differential expression analysis. (A) Volcano plot of the statistical result between allergic rhinitis (AR) and the control samples. The red color means up-regulated in AR group, the green color means down-regulated in AR group, and the black color means not significantly changed between the two groups. (B) The expression heatmap of the DEGs. (C) The distribution of DEGs across the genome.

We conducted differential gene expression analysis and identified a total of 31 DEGs between the AR and HC groups, including 17 high-expressed genes (MMP1, WFDC5, TRIB3, SULT1E1, CTH, ALOX12B, IGFBP3, LOC100132288, PCK2, SERPINB3, ALDH3B2, LOC284561, CRYZ, RBCK1, GCOM1, SLIT2, and ZNF83) and 14 low-expressed genes (SNRNP25, CDA, LOC100131512, SNX10, TSPYL5, HSPA1A, LOC645166, GTF2H2B, C17orf97, CECR1, LY75, LOC286434, GTF2H2, and EPDR1). DEGs were identified using the limma package with thresholds of p<0.05 and |log_2_FC|>0.585. The differential expression table was visualized with volcano plot, and the top 15 up- and downregulated gene expression between AR and control groups were displayed with heat map (Figure 2A and B). In addition, we showed the positional distribution of these DEGs in 23 pairs of human chromosomes (Figure 2C).

### Correlation analysis of the DEGs

**Figure 3:**
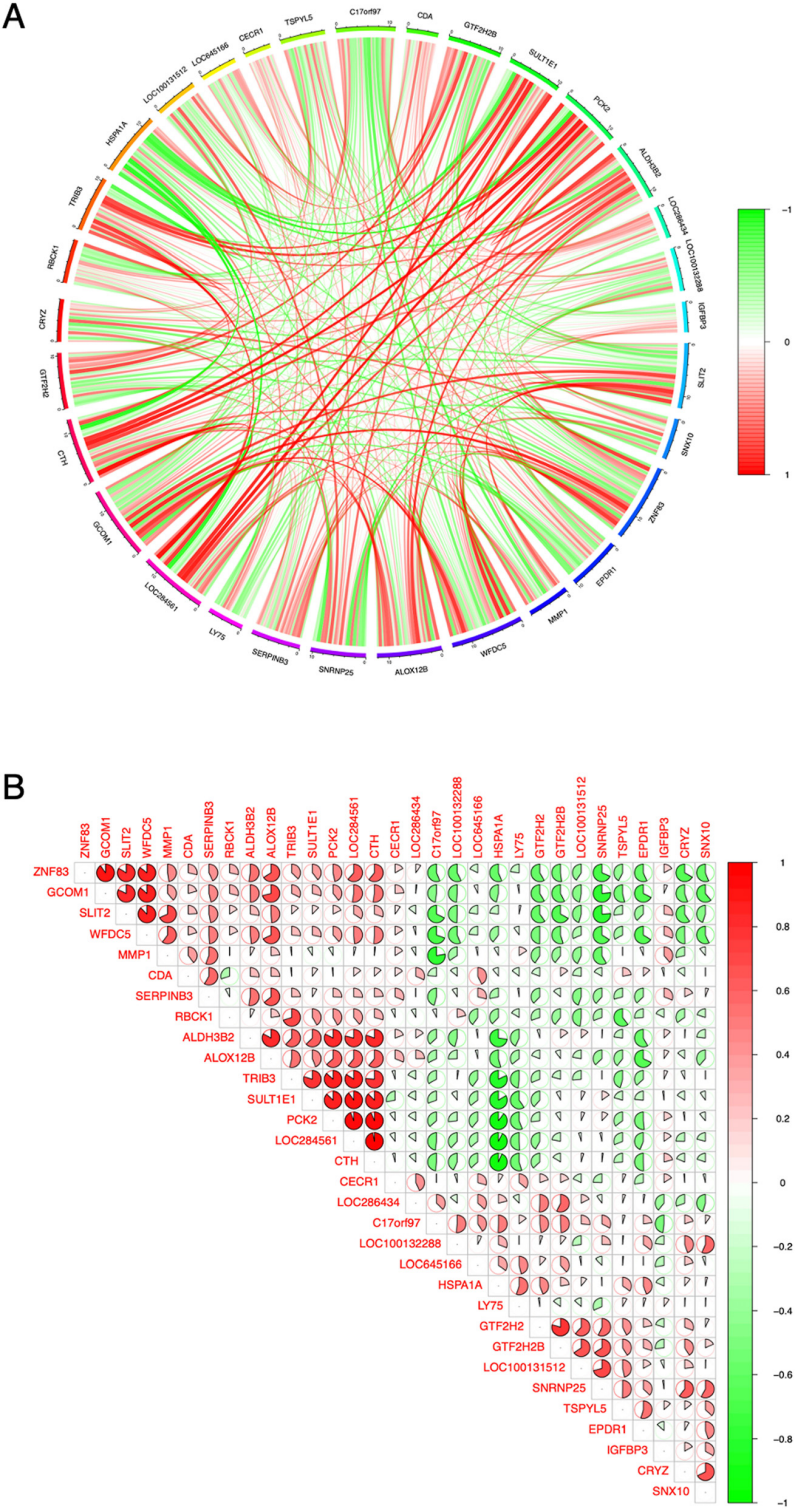
Correlation analysis of the differentially expressed genes (DEGs). (A) Circles representation of the correlation between DEGs. The red color represents positive correlation, and the green color represent negative correlation. (B) The pie representation of the correlation between DEGs. The red color represents positive correlation and the green color represents negative correlation.

To examine the potential relationships between the DEGs, we conducted spearman correlation analysis between the DEGs using the normalized expression matrix. The result showed that CTH has a positive correlation with LOC284561, PCK2, SULT1E1 and LOC284561, while HSPA1A’s expression is negatively correlated with CTH, LOC284561, PCK2, SULT1E1 and TRIB3 (Figure 3 A, B and Table S2).

### GO and KEGG enrichment analysis of the DEGs

**Figure 4:**
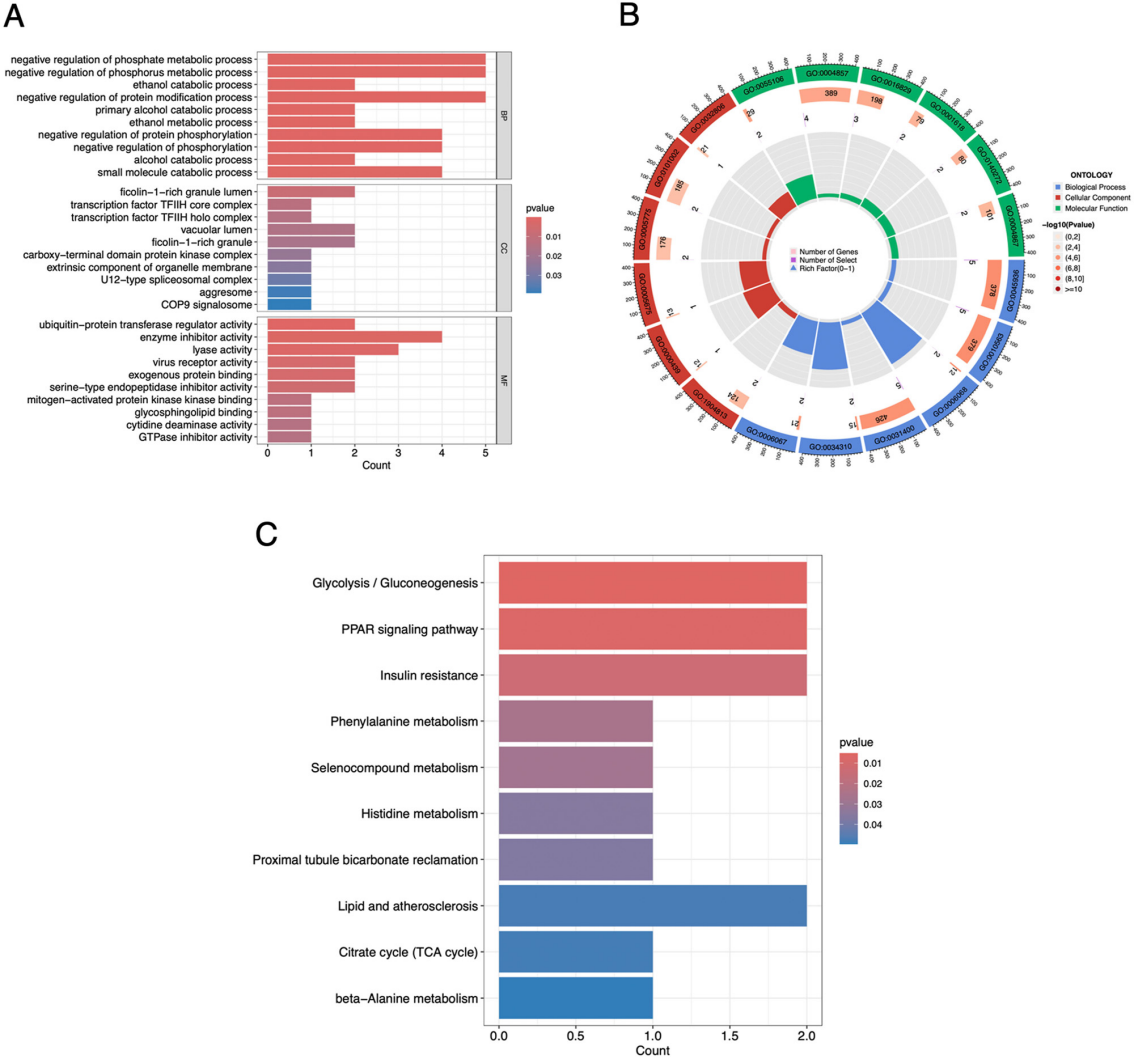
Functional enrichment analysis of differentially expressed genes (DEGs). (A, B) Top enriched gene ontology (GO) terms of each category. (C) Top enriched Kyoto Encyclopedia of Genes and Genomes (KEGG) pathway.

To gain inside the function of the DEGs, we conducted GO and KEGG enrichment analysis. We visualized the top 10 enriched terms of each GO category, showing that negative regulation of phosphate metabolic process, negative regulation of phosphorous metabolic process, etc. were enriched. Which implies that the DEGs’s function mainly related with those process (Figure 4A and B). KEGG enrichment shows that Glycolysis/Gluconeogenesis pathway and PPRA signaling pathway were significantly enriched (Figure 4C), both of the pathway is involved in glucose metabolic process. Taken together, the results imply that the main function of DEGs may be the inhibition of the glucose metabolism pathway.

### Immune infiltration analysis

**Figure 5:**
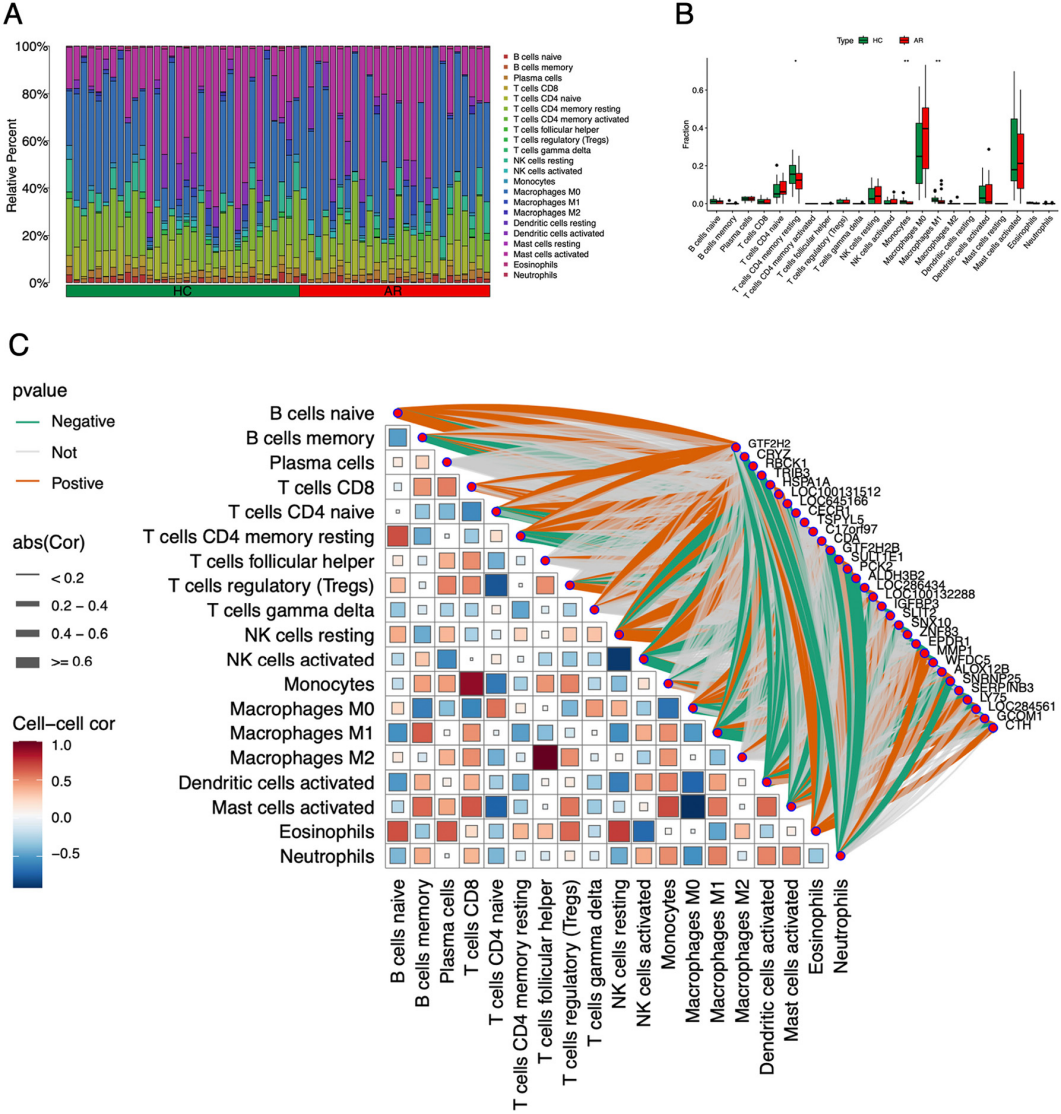
Immune infiltration analysis. (A) The infiltration levels of immune cells in each sample. (B) The statistical analysis of the immune infiltration levels between allergic rhinitis (AR) and the control groups. (C) The correlation between differentially expressed genes (DEGs) expression and immune cell infiltration levels. Between the cell-cell correlation the red color represents positive relationship, and the blue color represents negative relationship. Between the cell-gene correlation the orange color means positive correlation and the green color means negative correlation, and the width of the line is positively correlated with absolute value of the correlation coefficient.

To explore whether the immune infiltration levels of immune cells are difference between AR and control groups, we conducted immune infiltration analysis (Figure 5A). CIBERSORT’s deconvolution algorithm was used with 1,000 permutations. Wilcoxon rank-sum tests compared cell fractions between AR and controls (p<0.05). The analysis shows that T cells CD4 naive, monocytes and macrophages M1’s infiltration level between the two groups are significantly different (Figure 5B), and all of the three have a higher infiltration level in the control groups.

To examine the correlation between DEGs expression and immune cell infiltration levels, we conducted Spearman correlation analysis between gene expression level and immune infiltration level (Figure 5C). It showed that T cell CD4 naive’s infiltration level is positively correlated with GTF2H2 expression, Monocyte’s infiltration level is negatively correlated with CDA expression, and Macrophage M1’s infiltration level is negatively correlated with CRYZ.

### AR subtype analysis

**Figure 6:**
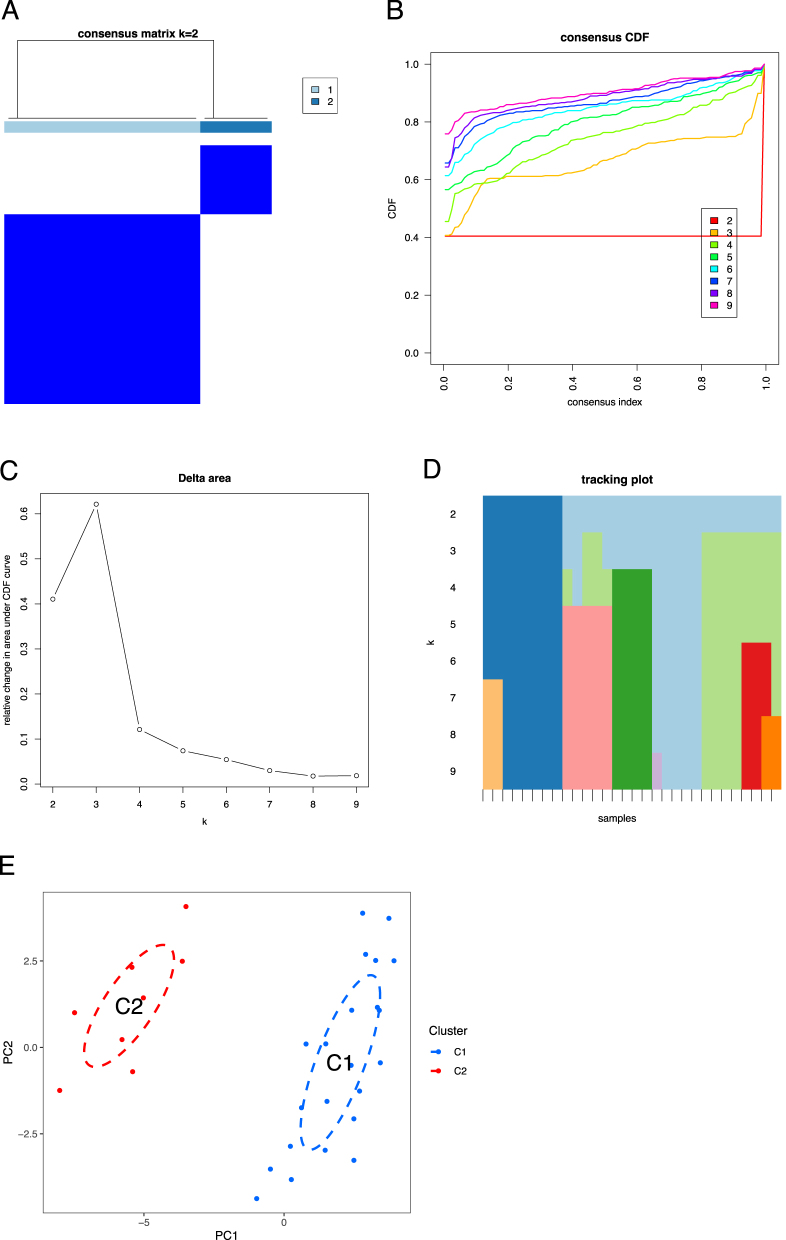
Allergic rhinitis (AR) subtype analysis. (A) The heatmap of the consensus clusters. The blue and white color represent how frequenctly pairs of samples cluster together across subsamplings. (B) The cumulative distribution curves. (C) The delta area plot, which shows the relative change in area under the cumulative distribution function (CDF) curve for successive k values. (D) The tracking plot, which shows how each sample’s cluster assignment changes as the k increase. (E) Principal component analysis (PCA) reveal significant differences between the two subtypes (C1 and C2).

To validate whether there are exist subtypes of AR, we performed consensus clustering analysis using the DEGs on all AR samples. Optimal cluster number (k=2) was determined by CDF area under the curve and consensus matrix. With CDF and area under the line, it showed that AR can be divided into two subtypes (Figure 6A–D), we named them as C1 and C2 subtype. And we validated the presence of the two subtypes with line dimensionality reduction analysis (Figure 6E).

**Figure 7:**
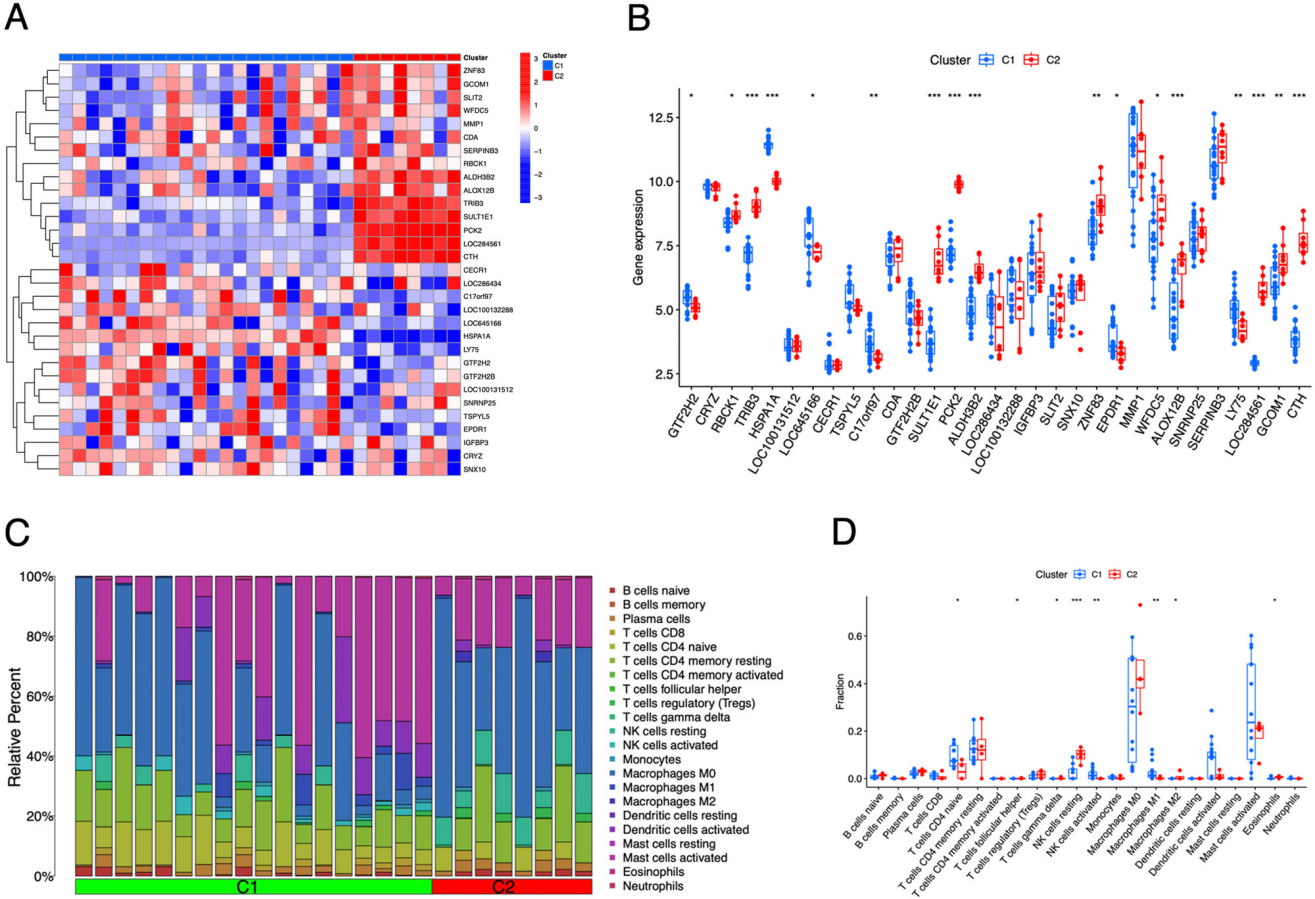
Immune infiltration analysis between allergic rhinitis (AR) subtypes. (A) The expression heatmap of the differential expressed genes (DEGs) between AR subtypes. (B) The boxplot of the DEGs between AR subtypes. (C) The infiltration levels of immune cells each sample. (D) The statistical analysis of the immune infiltration levels between the AR two subtypes.

Accordingly, we carried out differential expression analysis between the two subtypes and visualized the DEGs using heatmap and boxplot (Figure 7A and B). It showed that GTF2H2, RBCK1, TRIB3, HSPA1A, LOC64566, C17orf97, SULT1E1, RCK2, ALDH3B2, ZNF83, EPDR1, WFDC5, ALOX12B, LY75, LOC284561, GCOM1, and CTH are differentially expressed between the two AR subtypes. Immune infiltration analysis showed that follicular helper T cells, CD4 naive T cells, gamma delta T cells, activated NK cells, resting NK cells, M1 macrophages, M2 macrophages, and eosinophils infiltration levels are markedly changed between the two subtypes (Figure 7C and D).

### Machine learning–based screening of feature genes associated with AR

**Figure 8:**
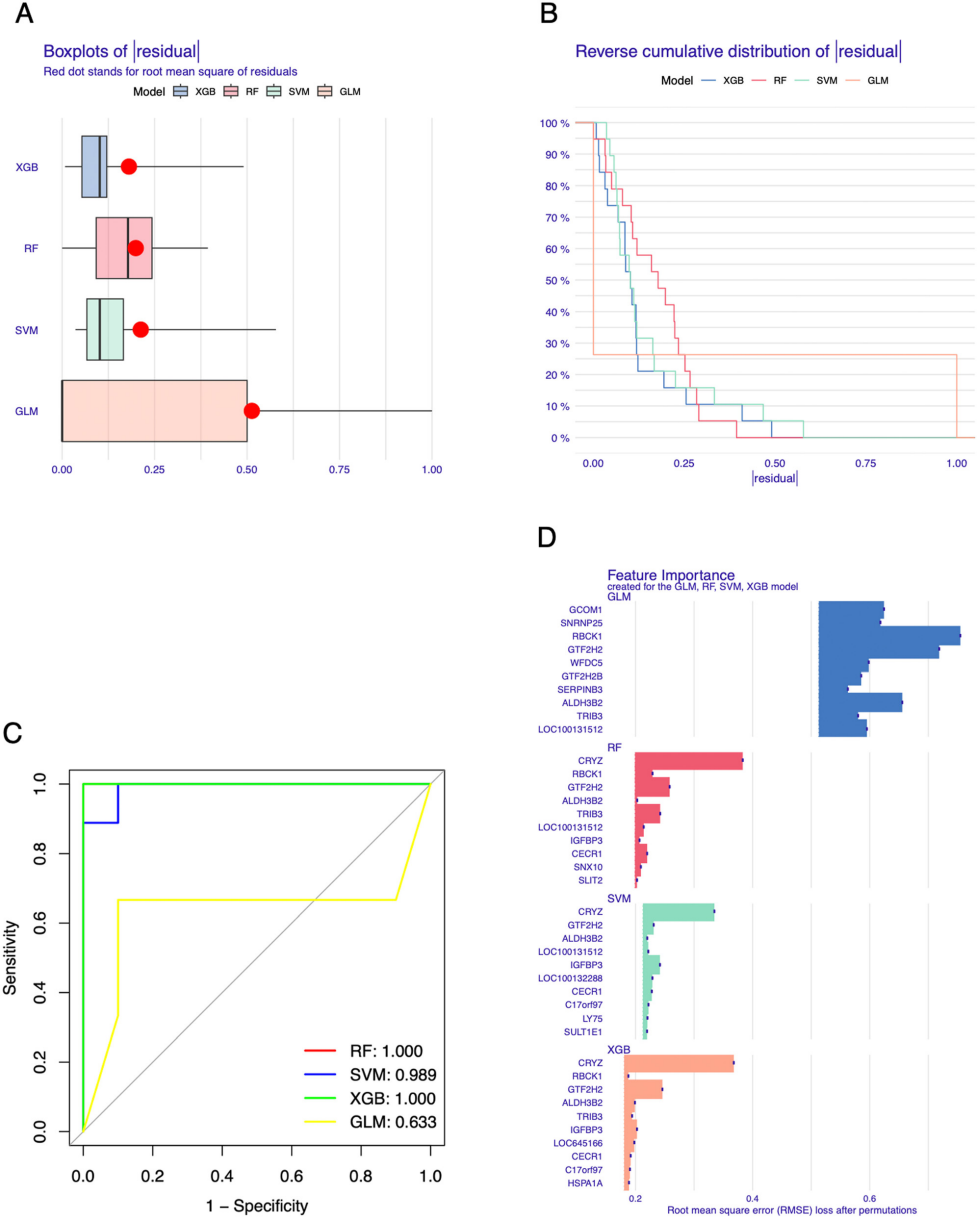
Screening feature genes for allergic rhinitis (AR) with machine-learning algorithms. (A) Boxplot of absolute residuals from four machine learning models. (B) Reverse cumulative distribution curve of absolute residuals for four machine learning algorithms. (C) Receiver operating characteristic (ROC) curve of four machine learning algorithms. The AUC score is located at the bottom right. (D) The top 10 most important features returned by RF, SVM, XGB and GLM.

To screen AR feature genes, we used four machine learning algorithms to screen AR feature genes from 31 DEGs. The residual distribution boxplot and cumulative distribution curve showed that the XGB model has the best stability (Figure 8A and B) and the ROC curve also showed that XGB has good classification ability (Figure 8C). Therefore, we chosed the XGB model and took the top five most important genes, LOC645166, ALDH3B2, IGFBP3, GTF2H2 and CRYZ, as the AR feature genes (Figure 8D).

### Validation of AR feature gene expression in the OVA-induced mouse AR model

**Figure 9:**
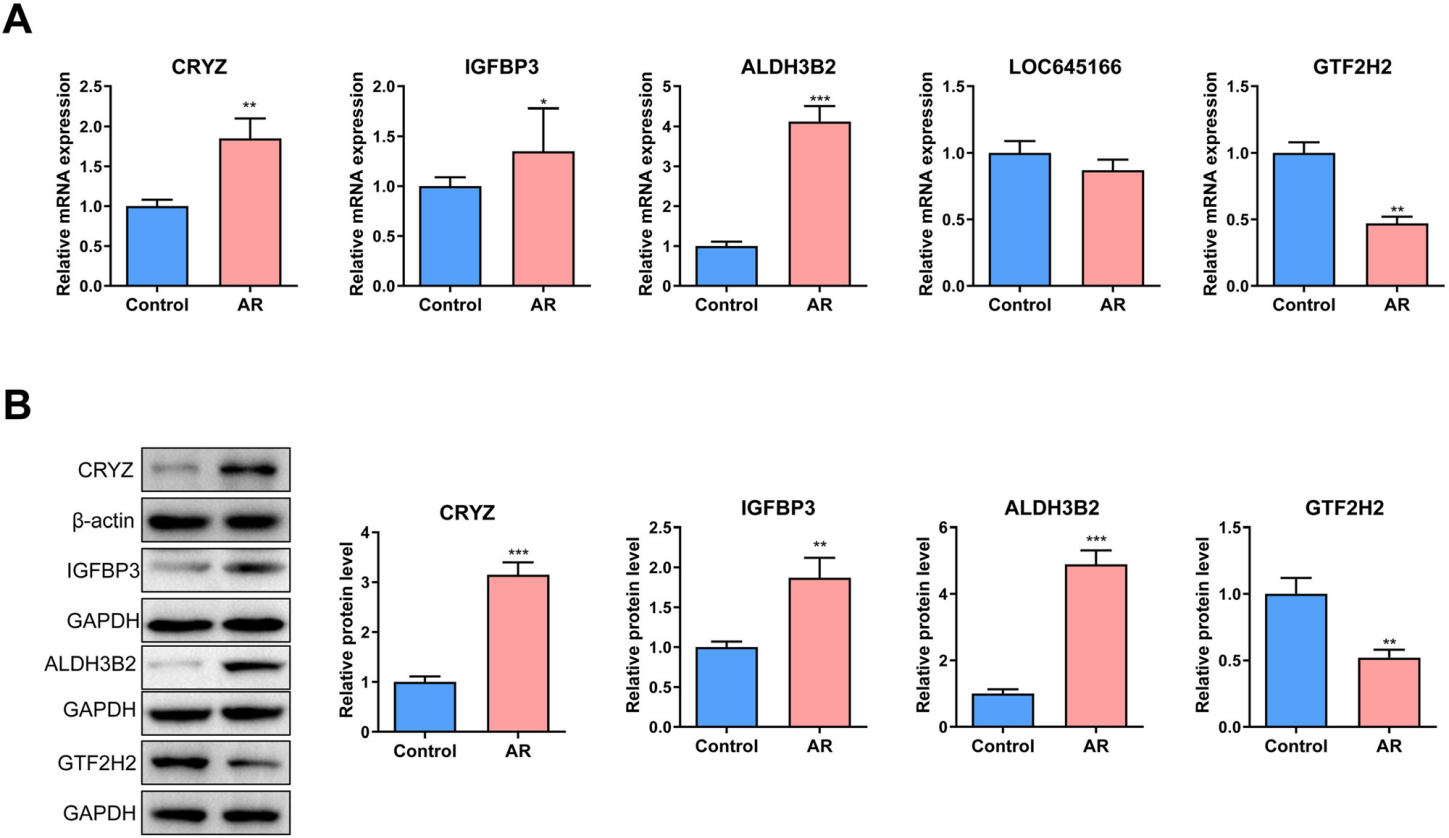
Validation feature genes expression in OVA-induced allergic rhinitis (AR) mouse model. (A) The RT-qPCR results show the relative mRNA expression levels of the five AR feature genes (CRYZ, IGFBP3, ALDH3B2, LOC645166, and GTF2H2) in the nasal mucosal tissues between the OVA-induced AR and the control groups. n=3. (B) The western blot results reveal the relative protein expression levels of the AR feature genes in the nasal mucosal tissues between OVA-induced AR and the control groups. n=3. *p<0.05, **p<0.01, ***p<0.001 vs. Control.

An OVA-induced mouse model of allergic rhinitis was established to validate the expression of key feature genes. Following OVA sensitization and challenge, nasal mucosal tissues were harvested for gene expression analysis. RT-qPCR results revealed that CRYZ (p<0.01), IGFBP3 (p<0.05), and ALDH3B2 (p<0.001) were significantly upregulated in the OVA-treated group, whereas GTF2H2 (p<0.01) was significantly downregulated (Figure 9A). Western blot demonstrated consistent expression trends for all genes except LOC645166, which because it is a non-coding gene (Figure 9B).

### Knockdown of ALDH3B2 reduced immune cell infiltration and activation in AR

**Figure 10:**
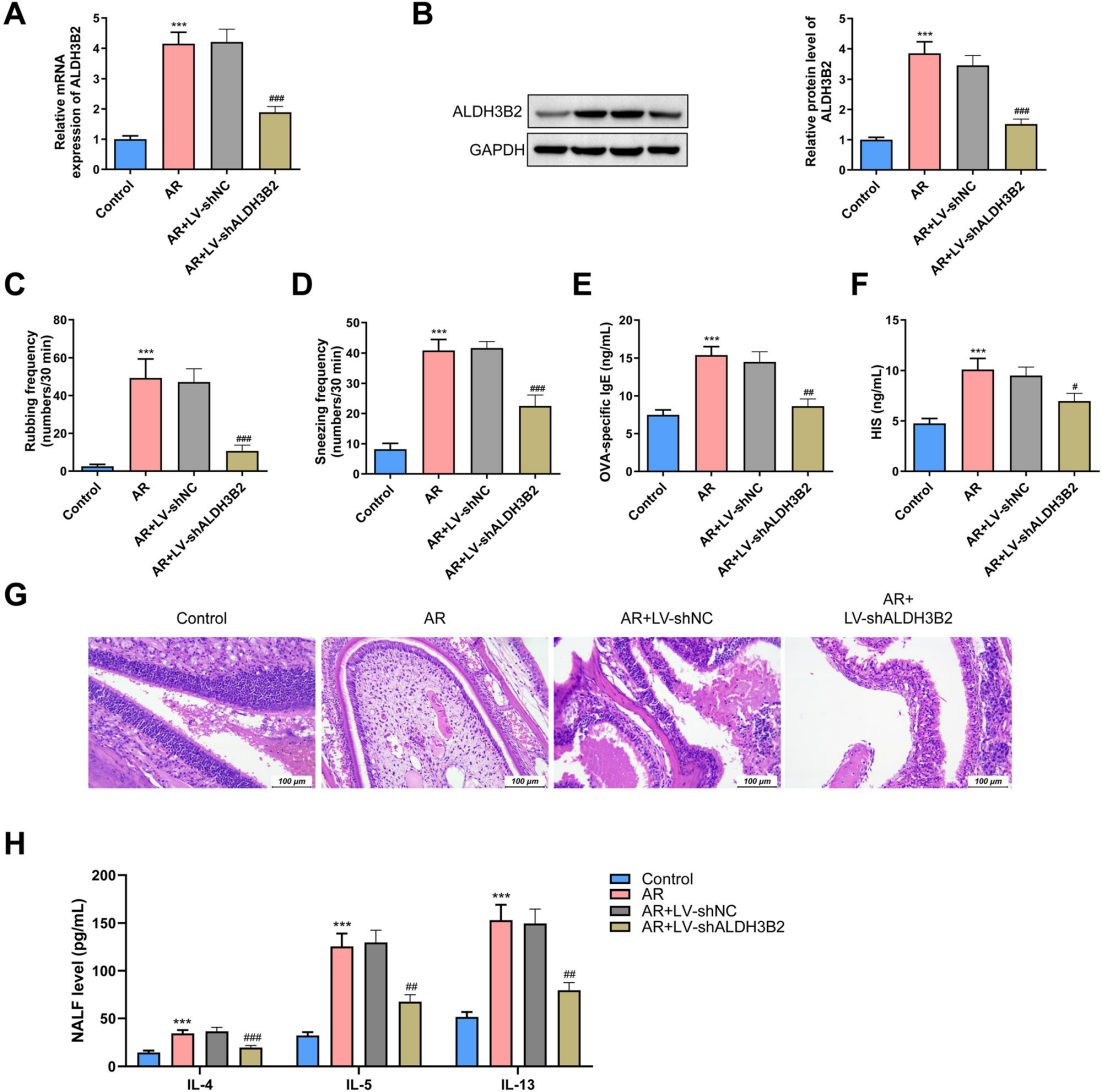
Knocking down ALDH3B2 alleviate immune cell infiltration and inflammation. LV: lentiviral, shNC: Negative control short hairpin RNA, shALDH3B2: ALDH3B2 mRNA target short hairpin RNA. (A) The relative expression of ALDH3B2 after tansfection of short hairpin RNA. n=3. (B) The protein levels of ALDH3B2 after transfection of short hairpin RNA. n=3. (C) The nasal rubbing of mice per 30 min n=8. (D) The number of sneezing of mice per 30 min n=8. (E) The level of OVA specific IgE in the serum. n=8. (F) The level of histamine in serum. n=8. (G) HE staining of the nasal mucosa tissue. n=3. (H) The contents of IL-4, IL-5 and IL-13 in the nasal lavage fluid are determined by ELISA. n=8. ***p<0.001 vs. Control; #p<0.05, ##p<0.01, ###p<0.001 vs. AR+LV-shNC.

To investigate the function of feature gene *in vivo*, we constructed a lentiviral vector expressing shALDH3B2 targeting ALDH3B2. After transfection, ALDH3B2 expression was markedly downregulated at both the mRNA (p<0.001) and protein levels compared to the AR+LV-shNC group in nasal mucosal tissues (p<0.001) (Figures 10A, 10B), as confirmed by RT-qPCR and western blot, respectively.

Following OVA challenge, typical AR symptoms-nasal rubbing, sneezing, and histamine release from mast cell – were significantly increased in the OVA-treated group (p<0.001). To assess the therapeutic impact of ALDH3B2 knockdown, we measured nasal rubbing frequency, sneezing episodes and the level of histamine and OVA-specific IgE in the serum. Mice transduced with the shALDH3B2 lentiviral vector exhibited marked reductions in all measured parameters compared to the AR+LV-shNC group (Figures 10C-10F) indicating that suppression of ALDH3B2 effectively alleviates AR-related inflammatory symptoms.

AR is an inflammation disease characterized by substantial immune cell infiltration in the nasal mucosa tissue. To assess whether ALDH3B2 knockdown affect local inflammation, hematoxylin and eosin (H&E) staining was performed on nasal tissue sections. The results showed a significant reduction in immune cell infiltration in the shALDH3B2-treated group compared to the AR+LV-shNC group (Figure 10G), indicating that suppression of ALDH3B2 alleviates AR-associated inflammatory cell recruitment.

Th2 cells are the predominant effector T cell subset in AR, differentiating from naive CD4^+^ T cells upon allergen exposure such as OVA. Once activated, Th2 cells secrete key pro-inflammatory cytokines-including IL-13, IL-5 and IL-4 - that promotes IgE production, eosinophil recruitment, and mucus hypersecretion. To determine whether ALDH3B2 knockdown affects Th2 activation, we quantified the content of IL-4, IL-13 and IL-5 in nasal lavage fluid (NALF). The results demonstrated that silencing ALDH3B2 significantly reduced the expression of all three cytokines compared to the AR+LV-shNC group (p<0.01, p<0.001) (Figure 10H), suggesting that ALDH3B2 may play a regulatory role in promoting Th2-driven inflammation in AR.

### Construction and validation of a diagnosis model for AR risk

**Figure 11:**
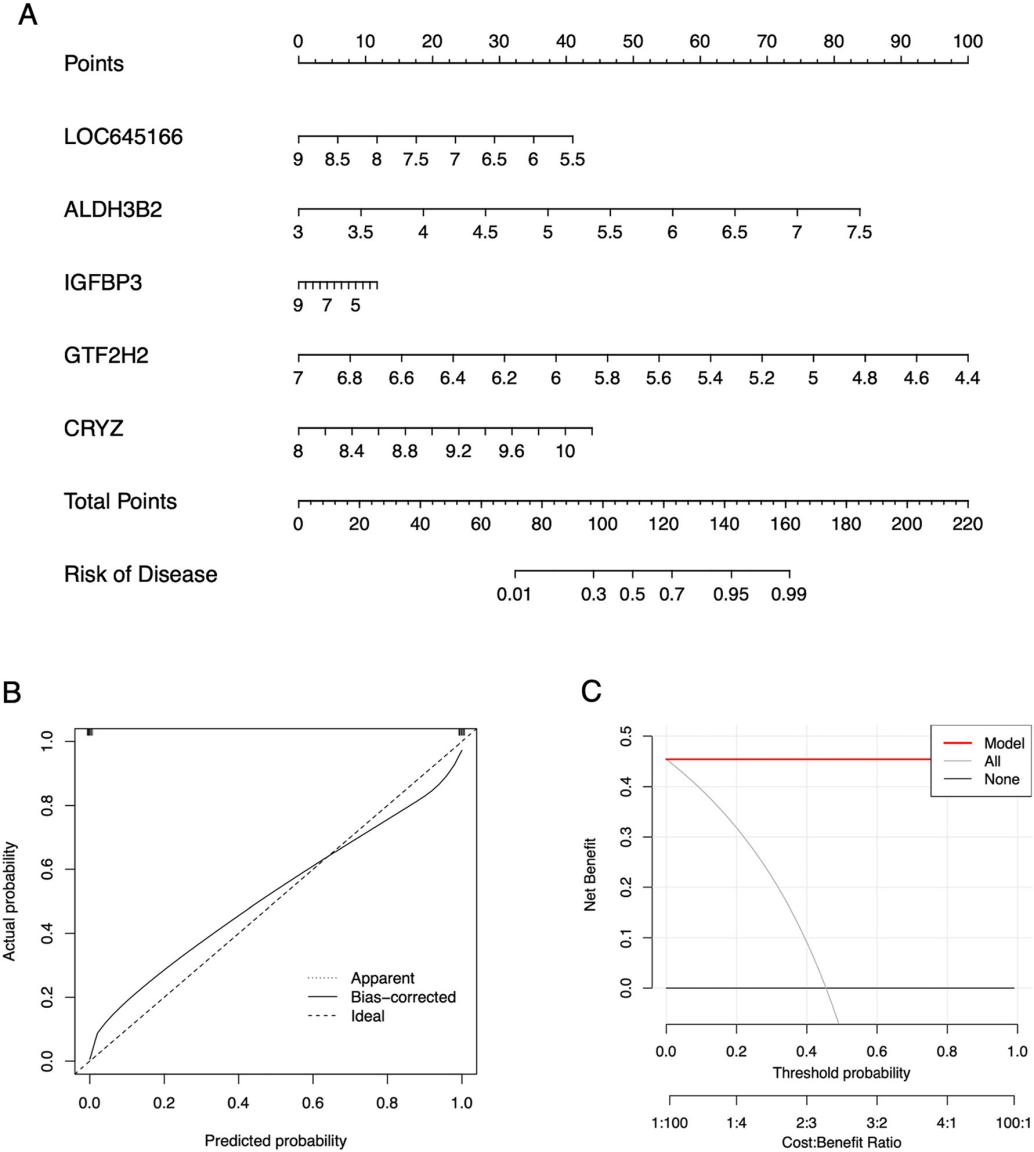
Using the allergic rhinitis (AR) feature genes to construct and validate the nomogram. (A) The nomogram model constructed with the expression of the AR feature genes. (B) The calibration curves of the nomogram. The x-axis represents AR onset probability and the y-axis represents the actual diagnosed AR. The dashed line represents predictions from the ideal model, and the solid line means the bias-corrected performance of the nomogram. (C) The decision curve for the nomogram. The x-axis represents threshold probability, this refers to the probability at which a patient would choose to act. The y-axis represents the net benefit, the trade-off between true positives and false positives, higher values indicate better clinical outcomes.

To predict the onset probability of AR, a prognostic model was constructed based on five AR feature genes – LOC645166, ALDH3B2, IGFBP3, GTF2H2 and CRYZ – identified using the XGB machine learning algorithm (Figure 11A). To assess the performance and clinical applicability of the model, both a calibration curve and a DCA were generated. The calibration curve demonstrated strong consistency between predicted and observed outcomes, while the DCA indicated favorable net clinical benefit across a range of the threshold probabilities (Figure 11B and C), supporting the robustness and predictive reliability of the model.

## Discussion

In the present study, we aimed to characterize immune infiltrates, molecular subtypes, and signature genes in AR. We found that AR patients exhibit significant alterations in specific immune cell populations (including T cells memory resting, Monocytes, and Macrophage M1) and can be stratified into distinct molecular subtypes based on DEGs. Furthermore, by employing multiple machine-learning algorithms, we identified and validated a set of five feature genes (including ALDH3B2, CRYZ, IGFBP3, GTF2H2, and LOC645166) that effectively distinguish AR from controls. A diagnostic nomogram constructed using these genes showed high predictive accuracy. Importantly, *in vivo* validation in an OVA-induced AR mouse model confirmed the differential expression of these genes and demonstrated that knockdown of ALDH3B2 significantly alleviated AR symptoms, highlighting its potential therapeutic role.

Over the past few decades, the global surge in allergic diseases has posed a substantial medical and socioeconomic burden. Biomarkers are valuable tools for precision medicine, as they provide clues about on the disease type, identification of therapeutic targets, and monitoring of therapy efficacies [29]. In clinical, the combination of clinical history and SPT/sIgE test remains insufficient for the precise diagnosis of AR [30]. Therefore, several studies have been conducted to study the the biomarkers for AR [31], [32], [33], [34]. Meng et al. found that polymorphisms in TSLP (rs189867) and IL1RL1 (rs3771180), differentially methylated of SLFN12, MUC4 and FOX3 and altered miRNA of miR-210, and miR-125 can be server as AR biomarkers [31]. Other biomarkers such as CD23 expressed on B cells also been reported [32]. Although gene expression biomarkers have been reported for AR, but those studies have concurrently investigated molecular signatures across multiple atopic conditions – including asthma and atopic dermatitis – using peripheral blood mononuclear cells (PBMC) [34] as a readily accessible surrogate for systemic immune profiling. During this study, an integrative analysis of AR transcriptome data derived from nasal mucosa tissue samples using four machine learning algorithms was performed. This approach led to the identification of five candidate biomarkers – LOC645166, ALDH3B2, IGFBP3, GTF2H2, and CRYZ – with potential relevance to AR pathogenesis.

The inflammatory response in the nasal mucosa of AR patients has been extensively characterized. The immediate response to allergen exposure is characterized by typical symptoms such as sneezing, rhinorrhea, and nasal congestion with 5–30 min [35]. These immediate symptoms are caused by immediate IgE mediated mast cell releasing of histamine. In the later phase inflammatory response characterized by flux into the nasal mucosa of eosinophils, basophils, and T cells expression Th2 cytokines such as IL-4, IL-5, and IL-13 [36]. By using deconvolution algorithms, we revealed that T cells CD4 memory resting infiltration levels in the AR groups was significantly down-regulated. This imply that CD4^+^ memory T cell differentiated towards Th2 cells. In the meanwhile, we also observed that macrophage M0 infiltration levels was up-regulated after AR onset, while monocyte infiltration levels were significantly down-regulated, which might suggest that in the inflammation tissue monocyte differentiated into macrophage has been accelerated. To validate the function of AR feature genes obtained in this research, we knocked down ALDH3B2 *in vivo*, an AR feature gene, it showed that immune cell infiltration levels and Th2 activation released cytokines were also significantly reduced. The diagnostic nomogram offered a translatable tool for AR subtyping. Targeting ALDH3B2 may benefit patients unresponsive to antihistamines, as it modulates pathways upstream of IgE (e.g., glycolysis; Figure 4C).

Rhinitis encompasses a heterogeneous group of disorders that share common nasal symptoms such as rhinorrhea, obstruction, pruritus, and sneezing, typically arising from inflammation or dysfunction of the nasal mucosa. Among the recognized forms, AR, infectious rhinitis, and non-infectious NAR represent three widely accepted clinical subgroups [37], 38]. While several studies have focused on the etiology and management of AR, comprehensive molecular stratification of rhinitis subtypes remains limited. To date, only two studies have attempted to define rhinitis subgroups at the transcriptomic level: one explored distinctions among AR, infectious rhinitis, and NAR using gene expression profiling [37], and the other focused exclusively on subclassifying AR using predefined inflammatory gene signatures [11]. In contrast, our study leveraged a broader, data-driven strategy by analyzing the entire set of DEGs between AR patients and healthy controls. Consensus clustering revealed two distinct and robust subtypes of AR, herein referred to as C1 and C2. Notably, immune infiltration analysis revealed that subtype C1 displayed significantly higher immune cell infiltration compared to C2, suggesting the presence of divergent immune landscapes and underlying immunopathological mechanisms. These findings imply that AR may not be a uniform clinical entity, but rather a spectrum of distinct molecular phenotypes with varying degrees of immune activation. This stratification holds potential clinical value: for instance, patients within the C1 cluster may exhibit heightened responsiveness to immunomodulatory therapies or biologics, whereas those in the C2 cluster might benefit from alternative therapeutic approaches targeting epithelial integrity or barrier dysfunction.

A potential concern regarding our cohort is the inclusion of Poly (I:C)-treated samples from the GSE51392 dataset. We acknowledge that Poly (I:C) can induce transcriptomic shifts; however, our study design intentionally incorporated these samples. The rationale was to explore the broad-spectrum molecular characteristics of AR under heterogeneous stimuli, including viral triggers known to exacerbate AR symptoms. This approach aimed to identify common pathways, such as altered glycolysis and phosphate metabolism, that may be relevant across different AR triggers. Importantly, the feature genes (e.g., ALDH3B2, CRYZ) identified through robust machine-learning algorithms (LASSO, SVM-RFE, RF, XGB) were subsequently validated in an OVA-induced AR mouse model, demonstrating their direct association with AR symptoms and confirming that these biomarkers are resilient to potential confounding effects from Poly (I:C) stimulation.

To date, more effective tools for predicting the onset of AR remain limited beyond conventional skin testing. To address this gap, we constructed a nomogram based on five identified AR feature genes, offering a novel approach for risk prediction and personlized management. This nomogram holds promise not only for forecasting AR onset in individual patients but also for evaluating therapeutic responses to pharmacological interventions. Although the use of male mice in this study minimized hormonal variability, future research should evaluate potential sex differences by including female subjects. Larger-scale validation studies are required to confirm the nomogram’s generalizability. While external validation with independent datasets will provide stronger evidence, the current scarcity of publicly available datasets containing all five feature genes presents a limitation. Ultimately, prospective clinical trials integrating these biomarkers will be crucial for further validation.

## Conclusions

In this research, five feature genes associated with AR – CRYZ, IGFBP3, ALDH3B2, LOC645166, and GTF2H2 - were obtained, which may serve as potential diagnostic biomarkers. Furthermore, we deciphered AR’s molecular heterogeneity, proposing ALDH3B2 as a therapeutic target and a machine-learning model for precision diagnosis.

## Supplementary Material

Supplementary Material

Supplementary Material
